# Effectiveness of Zinc Oxide Ointments Versus Non-Irritating Barrier Films in the Prevention of Incontinence-Associated Dermatitis

**DOI:** 10.3390/medsci14010086

**Published:** 2026-02-12

**Authors:** María Piedad García-Ruiz, Rosa Maria Torres Bautista, Maria Dolores Lopez-Franco, Agustina Orozco Cuadrado, Araceli Alarcon Juarez, Vicenta Nava Anguis, Francisco Pedro García-Fernández

**Affiliations:** 1 Advanced Practice Nurse in Complex Chronic Wounds, Jaén Norte Health Management Area, 23700 Jaén, Spain; 2 UGC Linares C “San José”, 23700 Linares, Spain; 3 Department of Nursing, Faculty of Health Sciences, University of Jaén, 23071 Jaén, Spain

**Keywords:** incontinence-associated dermatitis, non-irritating barrier films, zinc oxide, effectiveness, prevention, incontinence, skin protection, cost-effectiveness

## Abstract

Objectives: To evaluate the effectiveness of zinc oxide (ZnO) ointments versus non-irritant barrier films (NIBFs) in the prevention of Incontinence-Associated Dermatitis (IAD). Specific objectives included analyzing the effects of treatment over time, establishing when IAD appears in each group, and determining the safety and cost-effectiveness of both treatments. Methodology: A multicenter prospective cohort study was carried out in 10 social health centers in Spain. The final sample included 164 older institutionalized patients with urinary and/or mixed incontinence, divided into two cohorts (79 with ZnO and 85 with NIBF). Follow-up lasted for six weeks. Validated scales were used for data collection, such as the Categorization of Moisture-Associated Skin Damage (MASD) of the GNEAUPP (National [Spain] group for the assessment of pressure ulcers and chronic wounds) and the Visual Erythema Scale (VES). Results: The overall incidence of IAD was 20.7% in the sample during follow-up. No statistically significant difference in effectiveness was found between ZnO (27.8% incidence of IAD) and NIBF (32.9% incidence of IAD) in preventing IAD (*p* = 0.479). However, survival analysis suggested that the onset of IAD is delayed more in the NIBF group. No adverse events or side effects were reported attributable solely to the use of the products. When considering the total cost per process (including staff application time), ZnO ointment was EUR 0.02 more expensive per patient per day than NIBFs. Conclusions: Although both products have similar efficacy and safety, NIBFs delay the onset of IAD more than ZnO. In addition, despite a higher unit price, NIBFs are more cost-effective per procedure because of savings in nursing time during application and removal.

## 1. Introduction

Incontinence-Associated Dermatitis (IAD) is the most common form of Moisture-Associated Skin Damage (MASD), posing a significant challenge in healthcare due to its impact on quality of life and comfort among people in a situation of dependency [1]. The etiology of MASD is complex and multifactorial and is caused by the interaction of several factors that deteriorate the skin and reduce its barrier function [2,3,4] we could summarize the causal factors, according to the most relevant scientific literature [1,2,4,5,6,7] in the following points: excess moisture on the skin (this can come from incontinence (urinary, fecal, or mixed), sweating, exogenous solutions, wound exudates, effluents from stomas or fistulas, saliva, or mucus), action of chemical irritants (these include substances present in body effluents such as proteases and lipases in the feces, metabolites of drugs, and/or cosmetic products for inappropriate topical application) and activity derived from excessive and repeated cleaning and hygiene (due to physical irritation caused by certain inappropriate cleaning products or aggressive hygiene and/or drying techniques).

Etiologically, IAD is a complex and multifactorial condition caused by prolonged exposure to body effluents such as urine and feces. This exposure causes overhydration and maceration of the stratum corneum, making it vulnerable. It also alters the skin pH, thereby destroying the protective dermolipid barrier and facilitating the action of irritating fecal enzymes [1,2,7,8,9,10]. Skin injury is determined by numerous contributing factors such as exposure time, volume, quantity, content and type of irritant (the irritant capacity of water is lower for example than that of urine or liquid feces), pH of the moisture source (the increase in skin pH is a key mechanism, since the breakdown of urea into ammonia alkalizes the skin and destroys the dermolipid barrier), presence of pathogenic microorganisms on the skin or in effluents, skin conditions (integrity, turgor, hydration, etc.), associated mechanical factors such as friction and shear forces, the patient’s state of health (immunosuppression, diabetes, fever, malnutrition, etc.), extreme ages, and/or use of inappropriate or inappropriate occlusive absorbents or devices [1,2,4,5,6,7]

The management of IAD is based on a structured skin care program comprising three essential pillars: hygiene, hydration, and protection. Skin protection is crucial to prevent damage that results from constant exposure to irritating fluids [7]. In routine clinical practice, the most widely used barrier products for preventing IAD are zinc oxide (ZnO) ointment and non-irritating barrier films (NIBFs) [11].

ZnO ointment is valued for its anti-inflammatory, antibacterial, and healing properties, and for forming a robust physical barrier against moisture [12,13,14,15]. However, ZnO is formulated in oily bases that, while minimizing absorption, make it difficult to remove, thereby requiring friction and potentially damaging fragile skin [15,16]. On the other hand, NIBFs, developed in the 1990s, are acrylic polymers that form a transparent, durable, and waterproof film, and their key characteristic is that they do not contain alcohol, so they do not cause pain or stinging when applied to irritated or injured skin [17].

Despite the widespread use of these two treatments, the scientific literature has consistently pointed to the dearth of primary studies of high methodological quality that directly compare the efficacy and cost-effectiveness between ZnO ointments and NIBFs in the prevention of IAD [11,18]. The lack of robust evidence has led to the care implemented often being based on personal experience or opinion, rather than on standardized protocols. Some available comparative studies suggest that, although no significant differences in effectiveness were found, NIBFs may be more economically profitable than ZnO ointments. It is imperative to conduct robust research to quantify the relative efficacy of these products, establish evidence-based protocols, and optimize both clinical outcomes and cost-effectiveness of care.

Therefore, this study aims to address this knowledge gap by comparatively evaluating the effectiveness of ZnO ointments versus NIBFs in the prevention of IAD, in order to standardize care and improve patients’ quality of life.

## 2. Objectives

To assess the effectiveness of ZnO ointments versus NIBFs in the prevention of IAD. To analyze the effects of ZnO treatment and NIBFs over time, and to establish when the unwanted effect (IAD) appears first in each group. To determine the safety, side effects, and cost-effectiveness of both treatments studied.

## 3. Methods

Type of study. This research is part of a larger research project in which a multicenter prospective cohort study was carried out in 10 social health centers in several primary care districts in the provinces of Jaen and Cadiz, attending to the medical device used as prevention (zinc oxide—ZnO ointments—and non-irritating barrier films—NIBF) for moisture injuries associated with incontinence. This article presents the main objective of this research project. The cohort study is presented with two groups (cohort 1 treated with NIBF and cohort 2 treated with ZnO ointments). The same ZnO ointment and NIBF were used on all participants in each cohort. The zinc oxide ointment used is a dermatological protector that combines a barrier effect against moisture with a nourishing complex (amino acids, creatine, and almond oil) to regenerate the skin. Its zinc composition is 21%, and its antiseptic and anti-inflammatory formula prevents irritations due to incontinence, neutralizes odors, and strengthens the skin’s natural defenses without altering its pH [19]. The NIBF is composed of acrylic polymers that generate a transparent, durable, and water-resistant film on the skin, providing effective protection against fluids and friction. As it lacks alcohol, its application does not sting or is cytotoxic, guaranteeing a protective barrier whose durability can reach 96 h depending on the frequency of cleaning [20]. Both cohorts include patients with urinary incontinence and mixed incontinence, which were analyzed independently.

### Study Unit

- Study population. Patients treated in nursing homes in the Jaen-South Jaen, North Jaen, and Cadiz health districts, which care for dependent older adults, accredited by the Autonomous Community (ORDER of 5 November 2007 regulating the procedure and requirements for the accreditation of centers for older adults in a situation of dependency in Andalusia), with their own doctor and 24 h nursing care and who have urinary and/or mixed incontinence.
- Sample: Patients from the study population who met the inclusion/exclusion criteria agreed to participate in the study and who remained admitted from the date of the start of the study in the participating centers.
- Sample selection method: A non-probabilistic sampling of an intentional or convenience type was carried out, selecting all participants in the primary project from the beginning of the study until the sample size was completed.
- Inclusion criteria
  ○ Be over 18 years of age and have a life expectancy of more than 6 months from the start of the study.
  ○ Have urinary incontinence and/or mixed incontinence.
  ○ Use of absorbents on a continuous basis and as the only method for managing incontinence.
  ○ Previous absence of IAD.
  ○ Use regularly only one of the products under study: zinc oxide ointment or non-irritant barrier film.
- Exclusion criteria:
  ○ Patients with allergies to some of the study products.
  ○ Patients in a situation of terminal illness.
  ○ Presence of LPP, multifactorial lesions, or combined lesions prior to the start of the study in the gluteal, sacral, genital, or perigenital area.
- Withdrawal from study:
  ○ Need for bladder catheterization during the study.
  ○ Not being able to adequately carry out follow-up during the three months due to patient admission, transfer of residence, etc.
  ○ The patient’s own will.
  ○ Appearance during the study of the exclusion criteria.
- Sample size calculation: Patients were included according to the estimates made in the cohort study of the main project, which assumed an α error of 0.05, a statistical power of 80%, and considering that the IAD associated with patients with incontinence was 36% [21], a total of 110 patients (55 per cohort) would be needed. A 95% confidence interval will be established.

Variables analyzed: The following dependent variables were analyzed: sociodemographic variables (age, sex, associated comorbidities, pharmacological treatment, nutritional level, type of incontinence, degree of personal autonomy, risk of developing IAD, risk of developing pressure injuries, type of absorbent used and its frequency of change, as well as hygiene and skin protection methods) whose results have already been published [22]; presence and categorization of MASD, specifically la subcategory of MASD, IAD; appearance of side effects related to the application of the products of the study and profitability of both products.

Methods and instruments in data collection: Data collection was carried out through an ad hoc questionnaire and validated scales for those variables that had this option: Barthel Index, Perianal Assessment Tool, Braden Scale, Visual Erythema Scale, and Categorization of MASD proposed by the National Group for the Study and Counseling of Pressure Ulcers (GNEAUPP). The visual erythema scale consists of 5 items [23]: 0 (no erythema), 1 (little erythema, almost imperceptible), 2 (moderate erythema, pinkish skin), 3 (severe erythema, red or purple skin), and 4 (broken skin or superficial abrasion). The GNEAUPP MASD categorization consists of two categories [1]: Category I, where erythema appears without loss of integrity, a redness that may be unbleachable in a localized area usually subject to moisture. Within this category, lesions with mild-moderate pink erythema are classified as IA, and the IB classification is for intense dark pink or red erythema. Category II corresponds to erythema with loss of skin integrity. An open lesion with shallow depth, a red–pink bed, and usually macerated edges. It is classified as IIA when the erosion is less than 50% of the total erythema and IIB when the erosion is equal to or greater than 50% of the erythema.

Data analysis: A global descriptive analysis was carried out for this article. The qualitative variables are described using frequencies and percentages, and the quantitative variables are summarized by mean and standard deviation when normally distributed and by median and interquartile range when not. To analyze survival or time at risk, the Kaplan–Meier curve and Log-Rank test were used.

Ethical aspects: This study was approved by the Coordinating Committee on Biomedical Research Ethics of Andalusia (CCEIBA). The verbal and written consent of patients and/or relatives was requested, in accordance with the Declaration of Helsinki. The identification data were coded and treated confidentially, as indicated in Organic Law 3/2018, of 5 December, on Data Protection and ARCO Rights (Access, Rectification, Cancellation and Opposition). This study is also funded by the FIBAO (Foundation for Biomedical Research of Eastern Andalusia, Alejandro Otero) under Project code AP-0312-2022.

## 4. Results

### 4.1. On the Effectiveness of ZnO Ointments Versus NIBFs in the Prevention of IAD

Both study cohorts were homogeneous in terms of sociodemographic variables. This study analysed 164 participants from 10 social and health centres. The sample is mostly female (71.3%) with an advanced mean age of 85.7 years (range from 58 to 102 years) and a degree of severe dependence measured with the Barthel Index. The most prevalent personal histories were diabetes mellitus, renal failure, and arterial hypertension.

The incidence of IAD among participants with incontinence in this multicenter study was 20.7%. One in five participants during the six-week follow-up period developed IAD.

**Figure 1:**
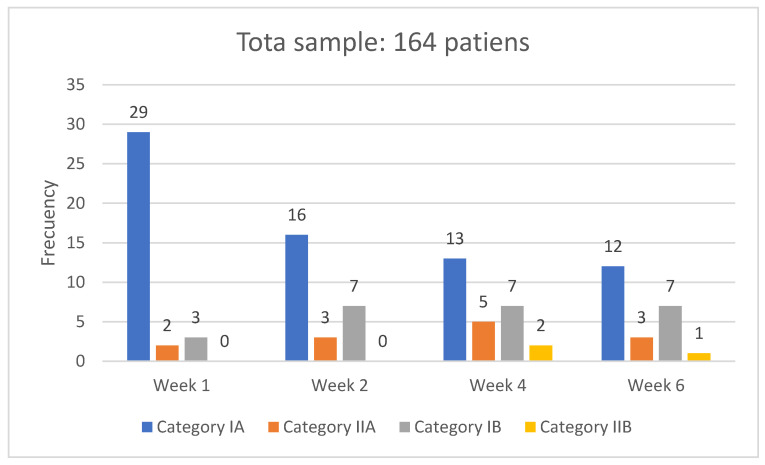
IAD incidence, frequency, and categorization.

Figure 1 shows the frequencies (N value) of IAD incidence and their categorization across the four measurements carried out throughout the 6 weeks of follow-up of the cohorts.

As shown, the highest incidence was observed in the first week, with the first measurement accounting for 20.7% in total, predominantly in category IA (17.7%). The incidence in the following weeks decreased by 15.9%, 16.5%, and 14%, corresponding to the measurements of the second, fourth, and sixth weeks of follow-up. Regarding the incidence of higher categorizations, such as IIB, only two cases were detected in week four and one case in week six.

**Figure 2:**
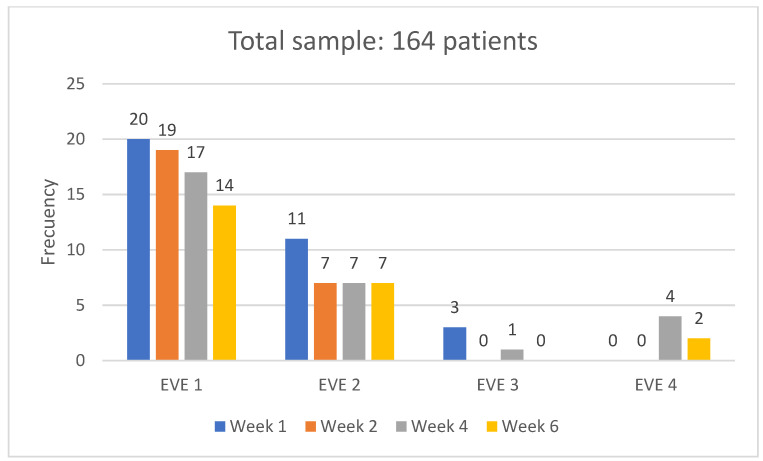
IAD frequencies according to the EVE Scale.

These data are in line with the measurement of the existence and categorization of erythema severity using the EVE scale, which is shown in Figure 2, where the maximum incidence detected was 12.2% for EVE 1 (little erythema, almost imperceptible) in the first measurement.

The incidence rate of IAD in the cohort using ZnO ointment was 27.8% (95% CI 18.0–37.7%), and in the NIBF cohort it was 32.9% (95% CI 22.9–42.9%), with the estimated relative risk being 1.18, although not significant (95% CI 0.74–1.89). This difference, assessed with the Chi-square test, also indicates no statistically significant association (*p* = 0.479), and the difference in means, adjusted for the Newcombe correction for independent samples, was −0.05 (95% CI: −0.19 to 0.09).

### 4.2. On the Effects of the Treatment over Time, and Establishing When the Unwanted Effect Appears First in Each Group

**Figure 3:**
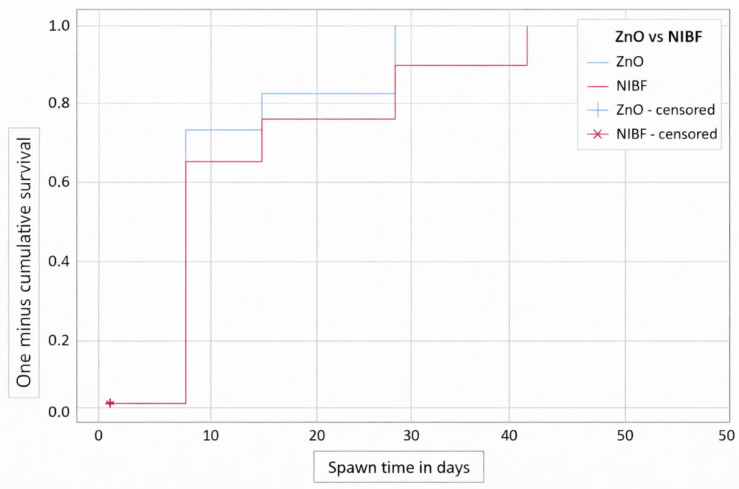
Kaplan–Meier curve for ZnO ointment use vs. NIBF. Note: Censored data represent patients who completed the follow-up period or dropped out of the study without developing Incontinence-Associated Dermatitis. Since all patients completed the study, the value is 0.

In Figure 3, we present the Kaplan–Meier survival curve, showing the cumulative probability of IAD with respect to the use of ZnO ointment versus NIBF. The NIBF cohort exhibits more events in the first days than the ZnO ointment cohort and shows a lower tendency to accumulate IAD events over time. However, this result, according to the Log-Rank test, is not statistically significant (*p* = 0.226).

**Figure 4:**
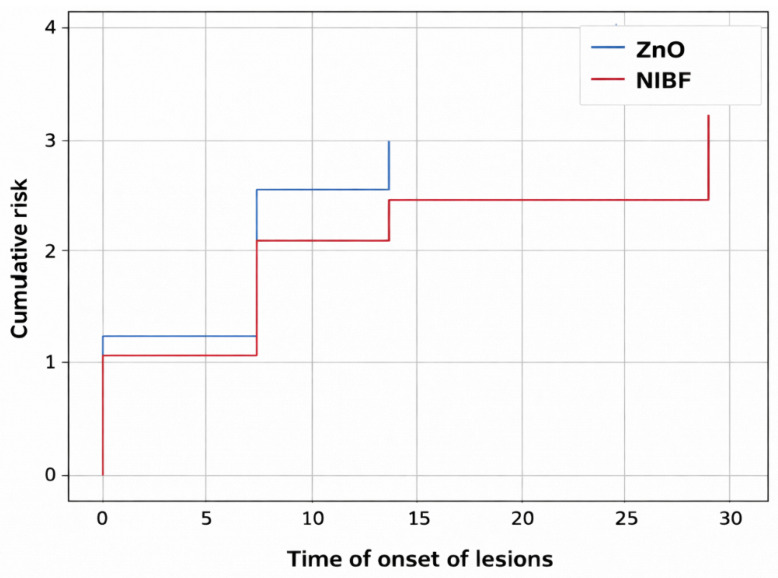
Kaplan–Meier curve for barrier methods (ZnO vs. NIBF), according to the risk function.

**Figure 5:**
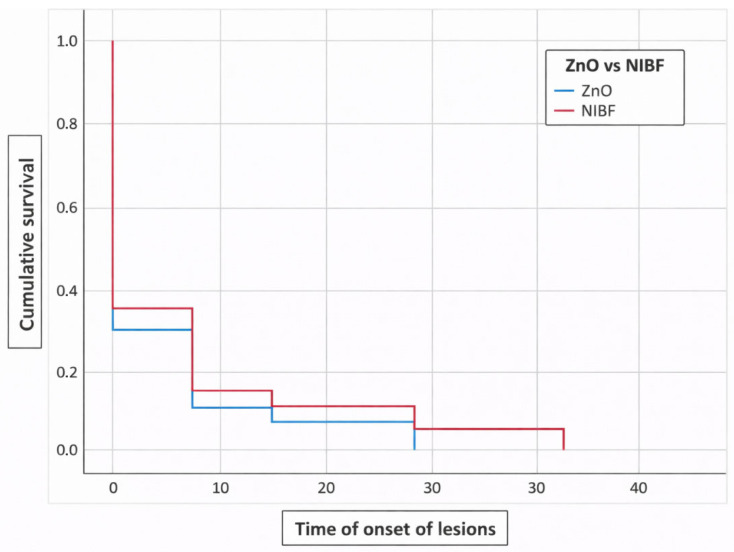
Kaplan–Meier curve for barrier methods (ZnO vs. NIBF), according to the survival function.

In Figure 4 and Figure 5, we observe survival and cumulative risk with respect to the use of ZnO and NIBF protective barriers.

When comparing the groups, a numerical trend was observed in the survival curves, with the NIBF group showing a slightly higher dermatitis-free survival rate at baseline compared to the ZnO group. However, this difference did not reach statistical significance (Log-Rank [Mantel–Cox] *p* = 0.226).

Table 1 presents the means and their 95% confidence intervals for survival time in both groups. As can be seen, both intervals overlap throughout the follow-up period, confirming the absence of statistically significant differences. Therefore, although we observed numerical differences in incidence, the current data do not allow us to conclude the clinical superiority of one intervention over the other in preventing Incontinence-Associated Dermatitis under the conditions of this study.

Finally, when assessing the association between the type of incontinence and the occurrence of IAD, we observed that in the urinary incontinence sub-cohort, 28.35% developed a lesion, and in the mixed incontinence sub-cohort, the incidence of injury was 32.63%. However, this difference with the Chi-square test is not statistically significant (*p* = 0.562), nor is it found in the analysis of the mean difference performed with the Newcombe correction for independent samples with a difference of −0.04 (95% CI −0.18 + 0.10) or with the relative risk being calculated at 0.31 (95% CI 0.06–1.49).

### 4.3. On the Safety, Side Effects, and Profitability of Both Treatments Studied

In the results presented above, the main complications derived from incontinence, such as pain, pruritus, burning, dermatitis, erythema, and burning, are specified, without independently analyzing the protection product applied in these incontinent patients (ZnO ointment or NIBF) and its relationship with the manifest side effects.

When analyzing the relative risk (RR) of ZnO ointment vs. NIBF on the occurrence of side effects, the result is 1.18 (95% CI 0.74–1.89); therefore, there is no statistically significant difference in attributing the occurrence of unwanted side events to one cohort more than another. However, these secondary events are attributed to IAD, which prevented these products only from the use of the products themselves. In no case was any adverse event or dermatosis related solely and exclusively to the product reported.

In a descriptive analysis of the collected cost-effectiveness data, we observed that 79 patients who used ZnO ointment used 29 complete tubes of ointment during the six-week study follow-up.

This means that the average number of uses for each tube is 116. The average time required for the 10 residences to apply this product after each hygiene treatment is 49 s, and the average time to remove the remains of the product from the previous hygiene treatment and apply it again on a new day is 86 s. Therefore, the total time required for application and removal is 135 s. Each tube of ointment costs EUR 11.132, so the treatment for 79 patients over 6 weeks was EUR 322.83.

In contrast, among the 85 patients in the sample who used NIBF for skin protection after hygiene, a total of 64 bottles were required.

Likewise, because the follow-up was six weeks and included daily application after hygiene, as in the previous cohort, the mean number of applications per bottle was 56. The time required to apply this product in the 10 residences averaged 21 s, and to remove it, 37 s, which is a total of 58 s. The cost of each bottle of this NIBF is EUR 13,64, with prices varying widely by point of sale.

Unlike the previous cohort that gave ZnO ointments to all those patients who used them from the project’s funding budget, for this cohort, it was the marketing company that provided all the necessary samples, so we cannot quantify the exact amount of each product, and the amount reported here is the average of 16 pharmacies consulted.

Therefore, the total cost for 85 patients with NIBF for 6 weeks is EUR 872.96.

Table 2 shows the cost per patient and cost per patient per day of each of the products used.

This is the raw cost data per product. In calculating the cost per process, the time required to apply each product and the caregiver’s salary are taken into account.

According to the 2024 VIII Dependency Agreement salary tables, a geriatric nurse assistant has a gross base salary of EUR 1132.07. Assuming they work an average of 20 days per month, their daily cost is EUR 56.60. If you work an average eight-hour workday, the hourly wage would be EUR 7.075, then it would be EUR 0.1179 per minute of work and EUR 0.0019 per second.

Table 3 presents the real cost, accounting for the per-unit cost and the per-hour cost of the worker when providing this care.

The application and withdrawal time for the ZnO product is 135 s, resulting in a cost of EUR 0.26 per patient per day. This is EUR 10.92 per patient, the cost of time worked during the 42 days of treatment.

While in NIBFs, it would be 58 s of application and removal. Therefore, it would be EUR 0.11 per patient per day, i.e., EUR 4.62 of time worked during the 42 days of study.

When applying the product, the collaborating workers referred to the advantage of NIBF over ZnO ointments that they could visualize the area and assess the skin to determine whether there is a lesion during each change of absorbent. As a disadvantage, not seeing the product exactly during its application, as it was transparent, caused them difficulty knowing if they had applied it to the entire area necessary or not, having sometimes given one or two more presses than would be necessary, to ensure that the entire area was covered.

On the other hand, the collaborating researchers detected excessive use of the NIBF in some cases by area or patient, probably due to what was previously commented on by the collaborating workers.

An abundant use of ZnO ointments was also detected in some cases, creating layers more than a millimeter thick as recommended. This is considered a disadvantage as it can excessively occlude the epidermis, reducing natural evapotranspiration as well as making it difficult to remove them later.

## 5. Discussion

### 5.1. About the Methodology

The sampling was non-probabilistic, with the sample size estimated assuming a 20% loss; even with 55 participants per group, the sample exceeded 79 in one group and 85 in another. A strength of this study is the number of participating centers, as 10 socio-health centers were selected from three Primary Care health districts in two provinces.

One potential limitation to consider is the deviation from the assumptions used in calculating the sample size. The incidence of dermatitis (20.7%) was lower than the a priori estimate (36%), reducing the total number of events, a key factor in the power of survival analysis. However, the final sample size of 164 patients, compared to the planned 110, a 49% increase, is understood to have mitigated this effect. Despite the lower event rate, the larger number of patients included allowed for a robust estimation of the survival curves, although the possibility of a type II error cannot be completely ruled out for very small effect sizes.

To control classification bias, and more specifically investigator bias, appropriate and validated measurement instruments were used, such as the GNEAUPP-developed MASD categorization (for the classification of one of the types of MASD, such as the DAI) and the EVE scale, and risk assessment of developing pressure injuries on the Braden Scale [24,25]. The researchers who would be in charge of carrying out the measurements for the evaluation of the appearance of lesions (MASD categorization of the GNEAUPP and EVE scale) were also specifically instructed to develop the ability and dexterity to categorize lesions correctly through the SECLARED program, and had to provide the diploma received on passing it in order to participate in the study.

On the other hand, the workers and researchers who measured the occurrence of IAD, erythema, and adverse reactions were unaware of which cohort each participant belonged to, maintaining this blindness throughout follow-up, although residual confusion cannot be excluded, despite the blinding of outcome evaluators.

Data processing was carried out using a computerized system of registration (RedCap) and statistical analysis (SSPS 24.0), which allowed the principal investigator to analyze the other dissociated from the names of the participants and the cohort to which they belonged, keeping this study blind. Although the principal investigator knew which group each participant belonged to at the beginning for the composition of the cohorts, when it came to analyzing the data, they were registered in RedCap by the other collaborating researchers.

Regarding the control of bias in the subjects studied, the observer bias or hawthorne effect, the patient knew what product was being applied to him because given the nature of each one it was not possible to hide it, but the patient had no way of influencing the result since the result did not depend on his will or influence whether he felt observed or not.

And finally, the exclusion and inclusion criteria were scrupulously applied, and the cohorts and sub-cohorts were balanced. In fact, although 11 centers were initially selected, the control of bias at one center could not be guaranteed, and it was withdrawn from the study without taking into account the data it provided, with ten centers remaining.

The statistical analysis was carried out in a multivariate and stratified manner. Third variables that could alter the result were taken into account, such as ZnO ointment brand and NIBF mark. To do this, each participating center was required to use the same ZnO and NIBF ointment. All participants voluntarily used these brands, so there was no variability in terms of percentages of ZnO, excipients, ether or alcohol content of the NIBF, prices, etc.

Other variables, such as the brand of absorbents and frequency of change of absorbents per day, hygiene method, and influence of sociodemographic variables, were specifically assessed in the statistical analysis, determining their possible statistical association with the dependent variable, and these results will be shown in another scientific article.

### 5.2. On the Effectiveness of ZnO Ointments Versus NIBFs in the Prevention of IAD and the Effects of Treatment over Time, and Establishing When the Unwanted Effect Appears First in Each Group

Our study sample reported an incidence of 20.7% in its first week of measurement, with most (17.7%) in category IA. This incidence is in line with the 6th National Study of the Prevalence of CDF in Spain, carried out by the GNEAUPP [26], which reported that, among the lesions reported in institutionalized patients, 17.5% were caused by moisture, with category IA being the most prevalent. In addition, the findings are similar to another study carried out on social health centers in Lleida in 2021 [27], which established the prevalence of IAD in institutionalized incontinent patients at 21.9%.

The overall prevalence of IAD varies across the literature, ranging from 5.2% to 50% [28]. In nursing home residents, the prevalence was 5.7% [7,29,30]. A total of 52.5% of individuals with fecal incontinence living in the community reported having IAD [31]. In patients with fecal incontinence, the prevalence can reach 50% [32]. In incontinent patients in intensive care units, the prevalence can be as high as 83% [32].

A 2018 RCT [18], which, similar to ours, was investigating the effectiveness of NIBF vs. ZnO, identified a slightly higher incidence percentage than our study, specifically 44%, which can be attributed to the situation in which the participants in his sample were. The greater severity or clinical instability of hospitalized patients makes them more vulnerable to developing the problem being investigated, which justifies the higher incidence rate of 44%. Those in our study were institutionalized in social and health centers without having any acute disease process that required hospital admission.

There is a contradiction in the effectiveness of the findings between studies. While the study by Baatenburg et al. (2004) [33] reported that NIBF was significantly more effective in improving skin condition and healing rates compared to zinc oxide oil, subsequent studies by Bliss et al. (2007) [34] and Goulart (2018) [18] found no statistically significant differences in efficacy between NIBP, ZnO, and control regimens (soap and water cleaning only).

The disparity could be due to methodological and contextual differences. The study by Baatenburg et al. [33] was in a nursing home and assessed improvement in skin condition, while Bliss et al. [34] was a multicenter study conducted in nursing homes in the U.S., and Goulart [18] was conducted in a general hospital with elderly patients hospitalized in Portugal, so the population and environment may influence the results.

Despite the lack of statistical significance in the most recent studies, Goulart’s study [18] showed a numerical trend toward a lower risk of IAD in the NIBF group compared to the control and ZnO groups, although this reduction was not large enough to be considered statistically significant. The advantage of NIBFs in terms of lower application frequency and shorter nursing time, as noted in Baatenburg et al. [33] and Bliss et al. [34], remains an important factor for cost-effectiveness and adherence, although the question focuses on direct clinical effectiveness.

Goulart’s study [18] also highlights the importance of risk factors for IAD beyond the barrier product used, such as adequate diaper size, stool consistency, nutritional status, and oxygen saturation, which should be monitored by nurses to prevent the problem.

Only Goulart’s study [18] used Cox regression to assess the effect of interventions (ZnO ointment and NIBF) on the risk of acquiring IAD. This study looked at the time to onset of IAD, which varied between groups. The group that received intervention II (NIBF) remained in the study longer (with an average of 10 days), and two patients in this group had signs of IAD at 250 and 260 days of follow-up. In contrast, the control group was the one that presented IAD in a shorter period (5.9 days). The risk of a patient in intervention II acquiring IAD was 0.58 times lower than that of the control group, although this difference was not statistically significant.

Our study shows with the Kaplan–Meier survival curve that the NIBF cohort had a higher number of events in the first days of treatment compared to the ZnO cohort, with this cohort having a lower tendency to accumulate IAD events over time, but similar to the Goulart study [18] these results were not statistically significant, according to the Log-Rank test in our case (*p* = 0.226).

Verdú et al. [35] in 2004 he carried out an RCT on 50 incontinent patients to measure the effectiveness of NIBF and ZnO ointments, but, although this work is considered a grey literature study (since the results were not published in any article except as a communication at a conference) we have found it very important to include them in the discussion in a secondary way through the study by Dr. García Fernández that reports in his systematic review of 2009 on the effectiveness of NIBFs in preventing skin lesions [36].

In summary, while one study suggests NIBF is superior to ZnO in effectiveness in improving the skin, two other studies failed to demonstrate a statistically significant difference in IAD prevention between these products and a basic hygiene regimen. This underscores the complexity of IAD and the need for a multifactorial approach to its prevention and management.

This highlights how important it is to take into account other variables influencing the appearance of IAD and that may be confounding variables when it comes to the prevention of the products studied, which is why we understand that this element is a strength of this doctoral thesis.

In the context of humidity lesions associated with ulcer exudate, there is an RCT published in 2005 and carried out in the United Kingdom [37] which compared the effectiveness of NIBPs vs. ZnO ointments with a 12-week follow-up on 35 patients and concluded that both products were equally effective (healing rate of 0.046 cm per week in the NIBP group and 0.039 cm in the zinc paste group) but NIBF was easier to apply and less time-consuming.

### 5.3. On the Safety, Side Effects, and Profitability of Both Treatments Studied

Our results did not show side effects or safety gaps regarding the use of one protective product or another (ZnO or NIBF ointment) since the adverse reactions found (pain, itching, burning, etc.) were attributable to the development of an IAD rather than to the use of a certain product itself.

One of the main considerations about ZnO is its opaque appearance, which prevents the skin below the application from being visible, requiring its removal for skin inspection [12,28]. The application of ZnO ointment is laborious, and its removal can be difficult and potentially painful for the patient [14,18,36]. The ZnO ointment adheres to the patient’s skin and may require mechanical friction to remove, which can cause irritation to already compromised skin [14,18]. Some ZnO products, especially viscous pastes, are difficult to remove [12,18]. The percentage of zinc oxide in ointments or creams influences this viscosity and the consequent difficulty of removal [15]. A 21% ZnO concentration was used in this study, but the need to determine the optimal ZnO concentration and its application at different stages of the lesion has been identified [11].

To determine possible limitations or interactions that the application of ZnO ointments may entail, it would also be necessary to consider the other ingredients. Some zinc oxide formulations may contain other ingredients such as menthol, chlorothymol, glycerin, lanolin, sodium bicarbonate, phenol, and thymol (in the case of Calmoseptine^®^ with 20% ZnO), or lanolin, petrolatum, and cod liver oil (in Desitin^®^ with 40% ZnO) [11]. It is not always clear whether the effectiveness or any side effects are due solely to the percentage of zinc or to the combination of ingredients [11]. In addition, other reviews have noted that ZnO products can interfere with the absorption of diapers [7]. However, mild zinc creams are considered a viable alternative, as they can be applied in a thin, transparent layer and are easier to remove than pastes [12] with an appropriate oil-based removal method, as they are fat-soluble products.

In contrast, NIBFs are transparent, allowing visualization of the underlying skin without the need to remove them for inspection [18,28,38,39]. There are studies that report that NIBFs do not cause stinging or pain when applied, which translates into greater comfort for the patient [17,18,36,39,40]. In general, it has been reported that NIBFs are non-cytotoxic products that are applied without observed adverse effects [15,18,39].

Although it is noted that barrier products may be a concern because they can block the pores of incontinence diapers and prevent moisture absorption, the breathable nature of NIBF mitigates this risk [28,38,41].

A 2016 Cochrane review, which explored different types of panty liners and hygiene products included in this project, noted that no data on pain due to the skincare product or procedure, or on adverse reactions such as skin irritation, rash, or allergic reactions, were reported in studies eligible for IAD [42].

If we compare both products in terms of comfort during application and removal, NIBFs have more bibliographic references supporting their ease of application and removal, and greater comfort for the patient than zinc oxide creams [14,17,18,36,39,40]. Difficulty removing ZnO can cause pain and increase manipulation of injured skin [17,18], presumably due to the improper application of an excessive amount and removal with water-soluble products; these difficulties could be mitigated with proper training and instruction on its proper use.

Our research did not report any complications in this regard, but we detected that, despite previous training on its use, a layer of ZnO ointment thicker than desired was applied in some cases, with the disadvantages we have already seen. Although the transparency of the NIBF is considered an advantage, allowing the examination of the skin without having to remove the product, it is precisely this invisibility that has, at times, caused, in this study, more pulses than indicated were applied, as the inability to see the product and even what skin surface was applied led workers to sometimes use more.

Regarding profitability, this research found that the cost per patient per day was 2.40 times higher for NIBF than for ZnO ointments. However, if we also take into account indirect costs, such as the time spent by geriatricians in applying ZnO ointments, the result is reversed, and the use of ZnO ointments is EUR 0.02 more expensive per day than NIBF. This study was designed from an institutional perspective, and although the process-based cost approach is a strength, some biases could be recognized that we must clarify. Although the NIBF material was provided by the industry, the authors state that the cost analysis was carried out independently using public reference prices, although these may vary depending on the location. On the other hand, the time estimates were described by the workers themselves who applied and removed the product, although there may be variability from one worker to another, which is why we talk about average time estimates.

These results are consistent with evidence from the literature suggesting that NIBFs may be more cost-effective than ZnO ointments, mainly due to reduced nursing time required for application and removal [7,11,17,18,28,36,39,43].

The cost-effectiveness of these products is not only evaluated by the direct cost of the material but also by the nursing time and ease of application and removal, which significantly impact the costs per process and the quality of care [36,39,40], and this indirectly contributes to safety by reducing the frequency of skin handling.

ZnO is frequently noted for its ease of availability and relatively low cost as a product in itself [13,18]; however, despite its low initial cost, it has been found in multiple studies that ZnO can generate significant indirect costs, such as when applying and removing it, with more work time and the application of oils for its removal [11,18,36,38,39,40].

The reduction in application and removal time for NIBFs makes them more profitable when analyzed by process. One review found that the use of NIBFs reduced the nursing time required for dressing removal by 10 min [36] and that the average cost per process for NIBF was EUR 76.13, compared to EUR 102.96 for ZnO (33% more expensive) [36]. The cost per process, including nursing time, is significantly lower with NIBF than with ZnO ointments (discharge), as reported in this study [37].

Despite promising findings on the cost-effectiveness of NIBs, there is a general consensus in the literature on the dearth of high-quality studies in this field [7,11,17,36,43]. Therefore, this study aimed to provide high-quality evidence using two homogeneous cohorts, thereby allowing us to generate extrapolable results.

## 6. Conclusions

- There were no statistically significant differences in the occurrence of side effects between the products (ZnO vs. NIBF).
- No statistically significant difference was found in the effectiveness between ZnO ointments and NIBFs for the prevention of IAD or vice versa; however, NIBFs maintain a longer survival and a lower cumulative risk during most of the study, i.e., the onset of an IAD is delayed more in the NIBF group than in the ZnO group.
- The side effects observed were attributed to the IAD, not prevented by the products, rather than to the products themselves, and in no case were adverse events related solely and exclusively to the product reported.
- NIBFs were found to be more expensive in the unit cost of the product than ZnO ointments. However, when considering the actual cost per process (including the time required for application and removal by healthcare personnel), NIBFs were slightly cheaper than ZnO ointments. This is because the application and removal of ZnO ointment is slower and requires more professional time than that of NIBFs.

## Figures and Tables

**Table 1:** Means, standard deviations, and confidence intervals for survival time.

	Means	St. Desv.	95% Confidence Interval	
			Lower Limits	Upper Limits
ZnO Ointments	11,455	1757	8012	14,897
Barrier films	14,500	2303	9985	19,015
Global	13,160	1505	10,210	16,110

**Table 2:** Cost per patient and cost per patient per day for each of the products.

	Cost Per Patient *	Cost Per Patient Per Day **
ZnO Ointments	EUR 322.83 All 29 Used Boats/79 patients = EUR 4.09 per patient.	EUR 4.09 per patient/42 days = EUR 0.10 per patient/day
Barrier films	EUR 872.96 All 64 Used Boats/85 patients = EUR 10.27 per patient.	EUR 10.27 per patient/42 days = EUR 0.24 per patient/day
Difference	2.40 times more expensive NIBF use vs. ZnO ointments	

* Cost per patient is the total cost divided by the number of patients in each cohort. ** The cost per patient per day is the cost per patient divided by the 42 days (6 weeks) of the follow-up period.

**Table 3:** Actual cost per process.

	Cost Per Patient Per Day + Cost Per Process of Application and Removal Time	Total Cost Per Patient over the 42 Days
ZnO Ointments	EUR 0.09 + EUR 0.26 = EUR 0.36	EUR 3.95 (cost per patient over the 42 days) + EUR 10.92 (cost of application and removal time over the 42 days) = EUR 15.01
Barrier films	EUR 0.24 + EUR 0.11 = EUR 0.35	EUR 10.27 (cost per patient over the 42 days) + EUR 4.62 (cost of application and removal time over the 42 days) = EUR 14.89
Difference	EUR 0.02 more expensive per day for the use of ZnO ointments compared to NIBFs	

## Data Availability

The original contributions presented in this study are included in the article. Further inquiries can be directed to the corresponding author.
